# Supplementary material for: Deciphering the regulatory genome of Escherichia coli, one hundred promoters at a time
Source: eLife. 2020 Sep 21;9:e55308. doi: 10.7554/eLife.55308 (PMC7567609; doi:10.7554/eLife.55308)
Supplement: Transparent reporting form [file elife-55308-transrepform.docx]

***eLife’s* transparent reporting form**

We encourage authors to provide detailed information *within their submission* to facilitate the interpretation and replication of experiments. Authors can upload supporting documentation to indicate the use of appropriate reporting guidelines for health-related research (see EQUATOR Network), life science research (see the [BioSharing Information Resource](https://biosharing.org/)), or the [ARRIVE guidelines](http://www.plosbiology.org/article/info:doi/10.1371/journal.pbio.1000412) for reporting work involving animal research. Where applicable, authors should refer to any relevant reporting standards documents in this form.

If you have any questions, please consult our Journal Policies and/or contact us: [editorial@elifesciences.org](mailto:editorial@elifesciences.org).

**Sample-size estimation**

- You should state whether an appropriate sample size was computed when the study was being designed
- You should state the statistical method of sample size computation and any required assumptions
- If no explicit power analysis was used, you should describe how you decided what sample (replicate) size (number) to use

Please outline where this information can be found within the submission (e.g., sections or figure legends), or explain why this information doesn’t apply to your submission:

To compute an appropriate sample size initial pilot experiments were preformed on the *lacZYA,* and *dgoR, marRAB,* and *relBE* operons and were compared to previous studies. It was found that the number of independent promoter variants and number of sequencing reads was sufficient to produce results that qualitatively found the same binding sites as previous studies and produced measurements of transcription factor DNA interactions that had a pearson correlation coefficient of 0.78 or greater. We discuss sample size, comparisons to previous results, and validation of sample size in this experiment by further sub-sampling the data in appendix 3.

- You should report how often each experiment was performed
- You should include a definition of biological versus technical replication
- The data obtained should be provided and sufficient information should be provided to indicate the number of independent biological and/or technical replicates
- If you encountered any outliers, you should describe how these were handled
- Criteria for exclusion/inclusion of data should be clearly stated
- High-throughput sequence data should be uploaded before submission, with a private link for reviewers provided (these are available from both GEO and ArrayExpress)

Please outline where this information can be found within the submission (e.g., sections or figure legends), or explain why this information doesn’t apply to your submission:

The transcription of regulatory regions for each gene were measured 12 times, once under each of the growth conditions considered in this study. The distribution of mutants in the DNA library was measured independently for each of these experiments. Each sample came from the same underlying library of promoter mutants. Mass spectrometry data was collected between 1 and 3 times for each pair of control and target sequences, and enrichment ratios for the proteins were calculated from the enrichment ratios of two or more peptides of that protein.

Sequencing data sets were excluded from the analysis if they had too few sequences (< 20000). Sequencing data sets were uploaded to the short read archive with accession no.PRJNA599253 and PRJNA60336, and can be found at the urls <https://www.ncbi.nlm.nih.gov/sra/PRJNA603368> and <https://www.ncbi.nlm.nih.gov/sra/PRJNA599253>. Discussion of growth conditions, and exclusion conditions can be found in appendix 1. Data availability is listed in the Methods-Data Availability section.

**Statistical reporting**

- Statistical analysis methods should be described and justified
- Raw data should be presented in figures whenever informative to do so (typically when N per group is less than 10)
- For each experiment, you should identify the statistical tests used, exact values of N, definitions of center, methods of multiple test correction, and dispersion and precision measures (e.g., mean, median, SD, SEM, confidence intervals; and, for the major substantive results, a measure of effect size (e.g., Pearson's r, Cohen's d)
- Report exact p-values wherever possible alongside the summary statistics and 95% confidence intervals. These should be reported for all key questions and not only when the p-value is less than 0.05.

Please outline where this information can be found within the submission (e.g., sections or figure legends), or explain why this information doesn’t apply to your submission:

99% confidence intervals are calculated for the effect of mutation on gene expression, and the results are described in the methods section. An outlier test is utilized to test for enrichment in mass spectrometry, with corrections for multiple hypothesis testing as outlined in Benjamini and Hochberg. These methods are described in the manuscript in Methods – Analysis of Sequencing Results and Methods – DNA affinity chromatography and mass spectrometry. P-values for displayed mass spectrometry results are shown in the supplementary file p-values for outlier test for mass spectometry data

Comparing the results of the Reg-Seq project to past results was done with a Pearson Correlation Coefficient (the *lac* CRP transcription factor binding site has an r value of 0.98, the *dgoR* CRP site has an r value of 0.90, the *mar*RAB site has an r value of 0.78, and the *relBE* site has an r value of 0.80). These results are displayed in Figure 3.

(For large datasets, or papers with a very large number of statistical tests, you may upload a single table file with tests, Ns, etc., with reference to sections in the manuscript.)

**Group allocation**

- Indicate how samples were allocated into experimental groups (in the case of clinical studies, please specify allocation to treatment method); if randomization was used, please also state if restricted randomization was applied
- Indicate if masking was used during group allocation, data collection and/or data analysis

Please outline where this information can be found within the submission (e.g., sections or figure legends), or explain why this information doesn’t apply to your submission:

No masking was applicable to this study.

- We encourage you to upload relevant additional data files, such as numerical data that are represented as a graph in a figure, or as a summary table
- Where provided, these should be in the most useful format, and they can be uploaded as “Source data” files linked to a main figure or table
- Include model definition files including the full list of parameters used
- Include code used for data analysis (e.g., R, MatLab)
- Avoid stating that data files are “available upon request”

Please indicate the figures or tables for which source data files have been provided:

All energy matrix models are available at the GitHub page for the project (<https://github.com/RPGroup-PboC/RegSeq>), and are archived with Zenodo at https://doi.org/10.5281/zenodo.3953312 . Code for data analysis is either performed through the MPAthic package or is uploaded to the GitHub above and is also attached as Source Code. Figure Data files are provided as attached files and are available on the GitHub.
